# High-intensity helical flow: a double-edged sword in coronary artery haemodynamics

**DOI:** 10.1098/rsos.242184

**Published:** 2025-08-06

**Authors:** Chi Shen, Mingzi Zhang, Hamed Keramati, Diogo Ferreira de Almeida, Susann Beier

**Affiliations:** ^1^ School of Mechanical and Manufacturing Engineering, University of New South Wales, Sydney, New South Wales, Australia; ^2^ Centre for Healthy Futures, Torrens University Australia, Sydney, New South Wales, Australia; ^3^ Virtonomy GmbH, Munich, Germany

**Keywords:** coronary artery disease, haemodynamics, computational fluid dynamics

## Abstract

The role of helical flow in human coronary arteries remains uncertain, yet its understanding promises unprecedented insights into atherosclerotic processes. In this study, we investigated the effects of helical flow and key haemodynamic descriptors in 39 patient-specific left coronary artery trees of the Automated Segmentation of Coronary Arteries (ASOCA) dataset: 20 non-stenosed and 19 stenosed. Absolute helical flow intensity *h*_2_ correlated with higher time-averaged endothelial shear stress in all vessel segments regardless of stenoses (p<0.05). In stenosed cases, this correlation was so prominent that the vessel area exposed to adversely low time-averaged endothelial shear stress reduced (less than 0.5 Pa,p=0.0001), and simultaneously, areas of adversely high time-averaged endothelial shear stress increased (greater than 4.71 Pa, p<0.05) coinciding with high *h*_2_ regions. This suggests that helical flow in coronaries is not always protective, as previously thought, because it not only mitigates low time-averaged endothelial shear stress associated with long-term plaque development and restenosis but also exacerbates adversely high time-averaged endothelial shear stress linked to increased plaque vulnerability and even acute events. Our findings redefined the current understanding of the role of helical blood flow in cardiovascular atherosclerotic disease processes.

## 1. Introduction

Helical flow (HF) naturally exists in vascular systems [1], characterized by a downstream and rotational motion. Studies on the aorta [2–4] and carotid bifurcations [5] have highlighted the favourable effect of HF on haemodynamics by reducing flow disturbances marked by a reduced luminal area exposed to adversely low endothelial shear stress (ESS) and its cardiac cycle time average (TAESS) [6], associated with a reduced risk of plaque progression [7,8] after disease onset [9]. Contrary to this, HF intensity is also known to be higher in curved and stenosed segments, which are common arterial regions of adverse clinical events. Specifically, in human-idealized and patient-specific coronaries, severe vessel curvature and torsion were both linked to increased HF intensity (sample size = 3) [10–12]. Severe curvature (often referred to as tortuosity [13]) has been linked to adverse clinical outcomes such as artery stenosis [14], spontaneous coronary artery dissection (SCAD) [15] and myocardial ischaemia [16,17]. This underscores a contradiction between the reported protective effects of high HF intensity and its vessel-specific occurrence [14–17].

Similar to human arteries, torsion positively correlated to HF intensity in swine arteries [18]. Moreover, in swine, high absolute HF helicity *h*_2_ correlated to higher ESS magnitudes [19]. Extremely high ESS has been linked to expansive remodelling [20], destabilization and rupture of plaque [21–23], thus driving major adverse cardiac events [24,25]. Additionally, HF *h*_2_ was found to be significantly higher in human coronaries compared with swine [26]. Therefore, HF studies on high ESS and TAESS are warranted. The relationship between HF and the extreme threshold of these haemodynamic quantities has never been considered in previous human coronary studies.

Moreover, stenosis was found to significantly increase the helicity intensity in idealized coronary bifurcations [11]. It is understood that in stenosed arterial regions, plaque continues to grow immediately downstream, adjacent to the existing stenosis [27,28], with high TAESS at the stenosis where the luminal diameter is most narrow and low TAESS downstream immediately adjacent to the stenosis regions [29,30]. An intricate relationship becomes apparent, which warrants further HF-specific studies in patient-specific stenosed human arteries.

Overall, given the important relationship between HF intensity and ESS/TAESS distributions, several studies have explored how HF is influenced by coronary artery geometries, aiming to establish a link between arterial geometry, HF and haemodynamics. These HF studies were either based on swine [18,19], which may not directly translate to human coronaries [26], non-coronary artery vessels [2–5], only considered the left main coronary bifurcations [11], idealized/modified vessel geometries [11,12] or a limited number (sample size = 3) of patient-specific coronary arteries without vessel-specific analysis [10].

Here, for the first time, we included the adversely high TAESS in the investigation of HF quantified by helicity intensity (*h*_1_ and *h*_2_) and their balance (*h*_3_ and *h*_4_). The haemodynamics and coronary geometrical effects were analysed in 39 (20 non-stenosed and 19 stenosed) left coronary trees to deliver a human, non-stenosed versus stenosed, patient-specific, whole trees and large sample-size consideration. Besides TAESS, we also considered the oscillatory shear index (OSI) and relative residence time (RRT) for haemodynamics. For the geometrical features, we considered the curvature, torsion and diameter. This will elucidate the conflicting associations between the reported favourable haemodynamic effect of high HF intensity while also coinciding with vulnerable arterial tree regions prone to adverse clinical events.

## 2. Material and methods

### 2.1. Patient-specific geometries

The open-source Automated Segmentation of Coronary Arteries (ASOCA) dataset contains 40 left coronary artery trees [31,32], 20 non-stenosed and 20 stenosed. Here, we excluded one case because of extreme stenosis (greater than 90%), whereby the limited spatial imaging resolution would introduce large haemodynamic computation uncertainties in line with other published work [9]. The dataset acquisition is described elsewhere [32]; briefly, the trees were reconstructed from computed tomography coronary angiography (CTCA) acquired via a GE LightSpeed 64-slice computed tomography (CT) scanner with an electrocardiogram (ECG)-gated retrospective acquisition protocol. The in-plane image resolution was 0.3–0.4 mm, and the out-of-plane resolution was 0.625 mm. Three experts independently segmented the images to derive a majority agreement for a high-fidelity dataset. The distal branches were trimmed at locations where the diameter was smaller than 2 mm due to the limited CTCA resolution. More details are provided in the relevant literature [31,32].

### 2.2. Computational fluid dynamics set-up

The left coronary artery trees were simulated using ANSYS CFX (ANSYS Inc., Canonsburg, PA, USA). The discretization was performed using ICEM-CFD, embedded in the ANSYS package (v. 2023R1, Canonsburg, PA, USA). A mesh sensitivity analysis was conducted before the simulations to ensure the computational models’ accuracy, efficiency and reliability. The blood flow was assumed to be incompressible and non-Newtonian using a Carreau–Yasuda fluid model [33]. A laminar blood model was used for simulations. The maximum Reynolds number was below 2000 for all cases at all time steps. Since patient-specific flow conditions were not available, an allometric scaling law was used to scale a standard velocity waveform, allowing for a physiologically relevant approximation [34], with the volumetric flow rate at the inlet calculated as follows [35]:


(2.1) $$Q=1.43d^{2.55},$$

where Q is the cycle-averaged flow rate and d is the mean diameter of the left main branch. For each bifurcation, a flow-split outflow strategy was applied to determine the flow rate in the distal branches [35],


(2.2) $$\frac{Q_{sb}}{Q_{mb}}={\left(\frac{d_{sb}}{d_{mb}}\right)}^{2.27},$$

where $Q_{sb}$ and $Q_{mb}$ are flow rates and $d_{sb}$ and $d_{mb}$ are the mean diameters of the side and main branch, respectively. The scaling law and flow split are effective due to their strong fit to *in vivo* data, improved by empirical adjustments to Murray’s law [35]. In stenosed arteries, minor haemodynamic differences were observed across varying stenosis degrees under rest conditions when using flow-split and multi-scale modelling approaches [36–38]. The artery wall was assumed to be rigid, and a standard no-slip condition was applied [39]. A steady-state simulation was performed for each model, and its results were used as the initial condition for the transient simulations of four consecutive cardiac cycles. The results from the fourth cycle were extracted for analysis to minimize transient start-up effects.

### 2.3. Coronary geometrical and haemodynamic descriptors

**Figure 1.**
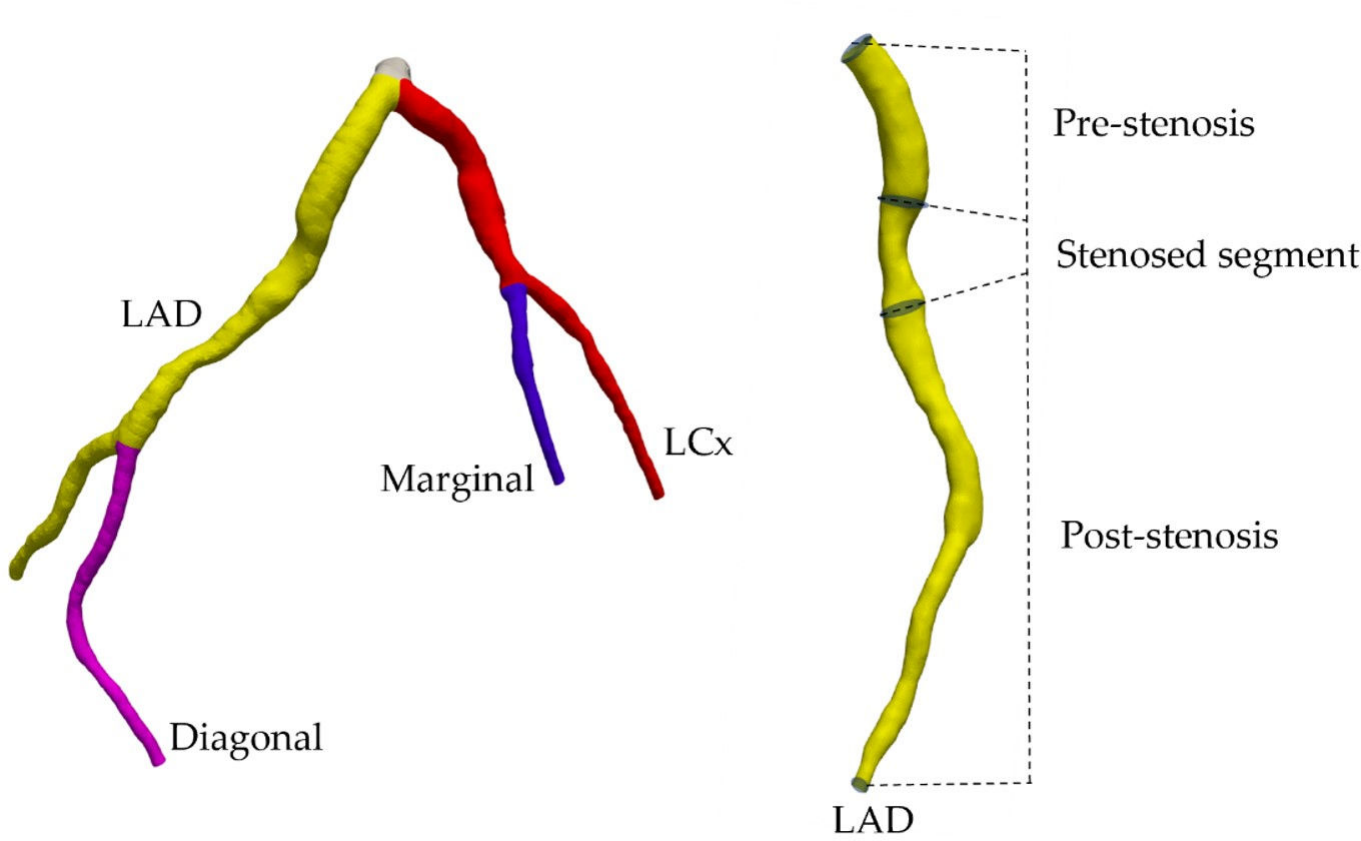
The breakdown of the left coronary tree into left anterior descending (LAD, yellow), left circumflex artery (LCx, red), diagonal (pink) and marginal (blue) segments (left). Vessel segments with stenosis were further analysed per pre-stenosis, stenosed and post-stenosis segments (right).

Both three-dimensional geometric and haemodynamic descriptors were extracted from each coronary tree, including the left anterior descending (LAD), left circumflex (LCx), diagonal and marginal arteries (figure 1, left). The stenosed regions were defined as the point of minimum luminal diameter extending two-vessel diameters upstream and downstream [40] (figure 1, right). The stenosed vessel segments were further subdivided into pre-stenosis, stenosed and post-stenosis regions for a detailed analysis of the stenotic effects (figure 1, right).

The geometrical descriptors considered include average absolute curvature (commonly referred to as tortuosity [13]), diameter and torsion, which were automatically calculated using in-house codes [41,42] using the Vascular Modeling ToolKit (VMTK),


(2.3) $$\kappa_{s}=\frac{1}{L}\int_{s_{1}}^{s_{2}}\frac{\left|c^{\prime }\left(s\right)\times c^{{\;}^{\prime \prime }}\left(s\right)\right|}{{\left|c^{\prime }\left(s\right)\right|}^{3}}ds,$$

where $c^{\prime }\left(s\right)$ and $c^{{\;}^{\prime \prime }}\left(s\right)$ are the first and second derivatives of the curve c, respectively, and L is the length of the curve. The torsion $\tau_{a}$ was calculated based on


(2.4) $$\tau_{a}=\frac{1}{L}\int_{s_{1}}^{s_{2}}\frac{\left|\left(c^{\prime }\left(s\right)\times c^{{\;}^{\prime \prime }}\left(s\right)\right)\cdot c^{{\;}^{\prime \prime \prime }}\left(s\right)\right|}{{\left|c^{\prime }\left(s\right)\times c^{{\;}^{\prime \prime }}\left(s\right)\right|}^{2}}ds,$$

where $c^{{\;}^{\prime \prime \prime }}\left(s\right)$ is the third derivative of the curve c.

The HF was visualized using the local normalized helicity (LNH) iso-surfaces, where right-handed and left-handed rotational flow patterns were colour-coded with red and blue, respectively. HF-based descriptors were used to quantify the density and rotational directions of HF [18,19]. Specifically, the cycle-average helicity (*h*_1_) and absolute helicity intensity (*h*_2_) were calculated, along with signed (*h*_3_) and unsigned (*h*_4_) helical rotation balance.

Adverse ESS distribution relates to endothelial dysfunction, promoting disease development [7,8]. Low ESS contributes to plaque progression and constrictive arterial wall remodelling [6]. Compared with low ESS, the area exposed to high ESS is related to expansive remodelling, increased plaque vulnerability with a higher necrotic core area and higher plaque burden [21,22], which can further lead to plaque rupture [23] and acute cardiac events [24]. In this work, we analysed the cardiac cycle averaged ESS (TAESS) to account for shear stress variations over the entire cardiac cycle, providing a more comprehensive measure to understand long-term effects. Several factors have been derived from TAESS. OSI quantifies the oscillation in the shear force direction at the vessel wall, where high OSI is associated with lipid accumulation and plaque erosion [43]. RRT indicates the regions with low TAESS exposure and high particle residence time. High RRT is related to atherosclerotic plaque calcification and necrosis [44]. Commonly used thresholds in the previous literature were adopted to define adverse values for TAESS (low, less than 0.5 Pa [45], and high, greater than 4.71 Pa [24,25]), high OSI (greater than 0.1 [46]) and high RRT (greater than 4.17 Pa^−1^ [44]). For each vessel segment, TAESS was reported as the average over the vessel lumen area. We also reported HF-based descriptors (*h*_1_, *h*_2_, *h*_3_ and *h*_4_), the normalized percentage of vessel area exposed to the adverse haemodynamic thresholds. All considered descriptors are shown in table 1.

**Table 1.** Definitions of helical flow and haemodynamic descriptors.

descriptors	equations	definitions
**helical flow-based descriptors**		
*h*_1_	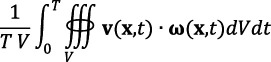	signed average helicity
*h*_2_	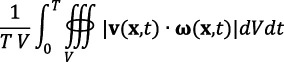	average helicity intensity
*h*_3_	$\frac{h_{1}}{h_{2}}$	signed balance of HF rotations
*h*_4_	$\frac{\left|h_{1}\right|}{h_{2}}$	unsigned balance of HF rotations
**LNH** localized normalized helicity	$\frac{v\left(x,t\right)\cdot \omega \left(x,t\right)}{\left|v\left(x,t\right)\right|\left|\omega \left(x,t\right)\right|}$	visualization of the helical flow
**ESS-based haemodynamic descriptors**		
ESS endothelial shear stress	$n\cdot {\vec{\vec{\tau }}}_{ij}$	shear stress along the vessel wall
TAESS time-averaged ESS	$\frac{1}{T}\int_{0}^{T}\left|n\cdot {\vec{\vec{\tau }}}_{ij}\right|dt$	the cardiac cycle averaged ESS
OSI oscillatory shear index	$\frac{1}{2}\left(1-\frac{\left|\int_{0}^{T}\tau_{w}dt\right|}{\int_{0}^{T}\left|\tau_{w}\right|dt}\right)$	representation of the change of the ESS vector from a predominant flow direction
RRT relative residence time	$\frac{1}{\left(1-2OSI\right)TAESS}$	the residence time of elements in the blood adjacent to the wall

*T*, cycle period; *V*, volume of interest; 𝐯(𝐱,*t*), velocity field at the location 𝐱 and time t; ω(x,t), vorticity; ${\vec{\vec{\tau }}}_{ij}$, endothelial shear stress tensor; **n**, normal vector at the vessel wall, $\tau_{w}$, shear force at the vessel wall.

### 2.4. Statistical analysis

Statistical analyses were performed using the Python Statsmodels package, v. 0.14.2. The normality of distribution was assessed with the Shapiro–Wilk test. Continuous variables with a normal distribution are presented as mean ± s.d., while non-normally distributed variables are presented as median and interquartile range. For comparisons between non-stenosed and stenosed groups, Welch’s *t*-tests were used for normally distributed variables and the Mann–Whitney *U*-test for non-normally distributed variables. Spearman correlation coefficients, ρ, and the regression *p*-value were used to evaluate the correlations between HF-based and haemodynamics, geometrical and HF-based, and geometrical and haemodynamic descriptors. To avoid type I error in the statistical hypothesis testing, *p*-values presented in this work were adjusted with the Bonferroni–Holm correction, with a *p*-value after adjustment less than 0.05 considered statistically significant.

## 3. Results

### 3.1. Comparisons between non-stenosed and stenosed groups

**Figure 2.**
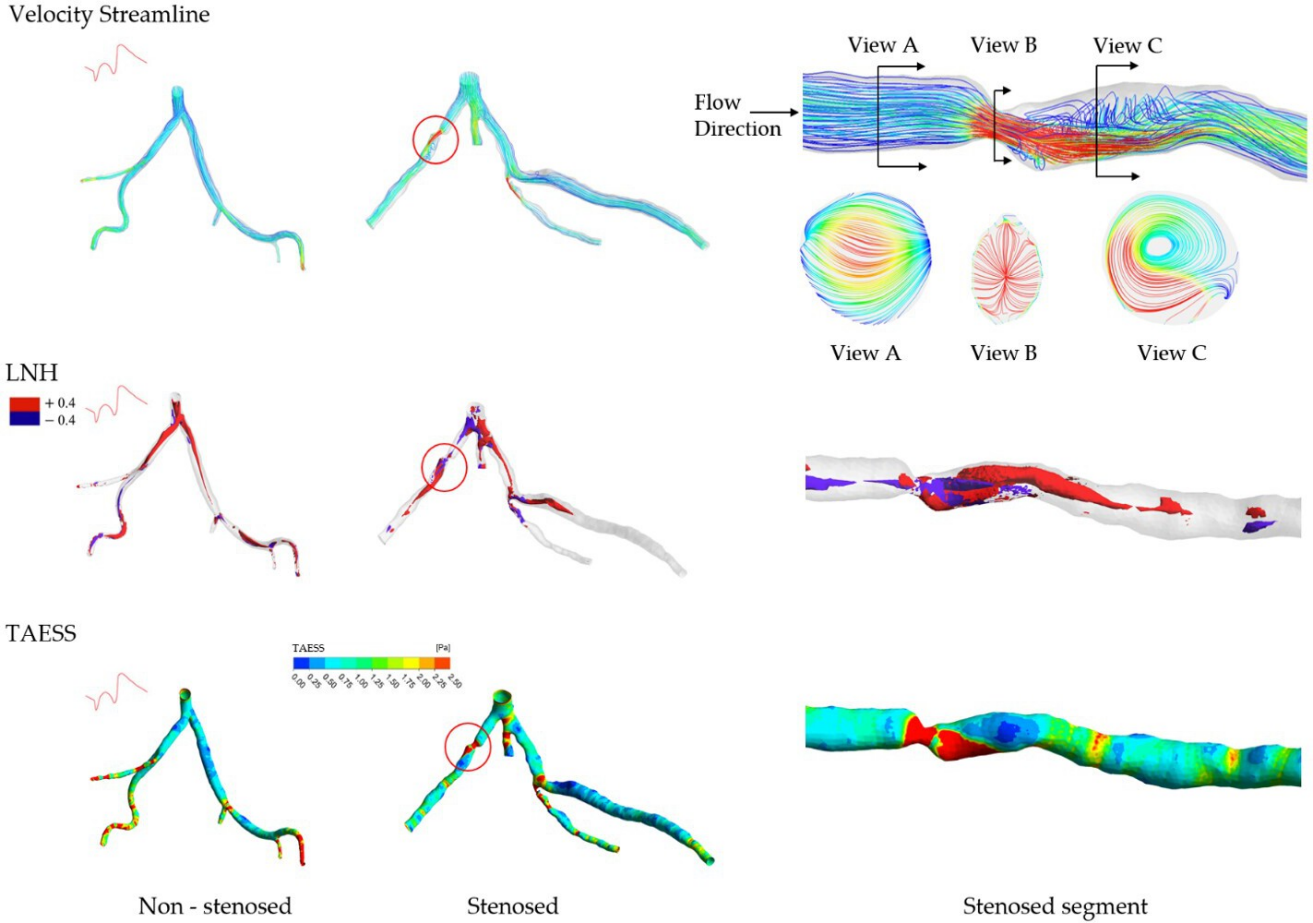
Blood flow characteristics in a patient-specific coronary artery tree: one non-stenosed case (left), one greater than 70% stenosed case (middle), and a zoomed-in view of the stenosed segment (right) highlighted by the red circle. Examples of time-averaged velocity streamlines (top), time-averaged localized normalized helicity (LNH) (middle) and time-averaged wall shear stress (TAESS) contour map (bottom) are shown. Disturbed flow patterns were evident in the stenosed regions and downstream, characterized by jet flows and recirculation. The streamlines captured in the arterial cross-sections (views A–C) manifest vortex formation proximal (A), within (B) and distal (C) to the stenosis. No vortex structures were observed proximal to or within the stenosed region (views A and B), whereas vortices were observed downstream of the stenosis (view C), due to blood flow recirculation. More intense and imbalanced HF patterns occurred in the stenosed segments.

Comparing the general haemodynamic distributions in artery trees from non-stenosed and stenosed groups, the existence of stenosis affected local haemodynamics, as shown in figure 2. Disturbed flow was evident in the stenosed regions and downstream of the stenoses. Jet flows developed at the stenosis with high TAESS distributions. Recirculation appeared immediately after the stenosis, where more intense and imbalanced HF patterns were also observed. The streamlines captured in the arterial cross-section indicate the presence of vortex structures downstream of the stenosis due to blood flow recirculation (figure 2, view C). No vortex formation was observed proximal to or within the stenotic region (figure 2, views A and B). The evolution of vortex structures at important time points, such as peak flow, throughout one cardiac cycle is depicted in the appendix, figure 5. Both low and high TAESS were downstream of the stenosis (figure 2). Here, the direct comparison between segments with and without stenosis cannot be conducted since the non-stenosed and stenosed arteries were not from the same patient. Thus, a vessel-specific comparison of the stenosis-free segments from both groups was applied.

**Figure 3.**
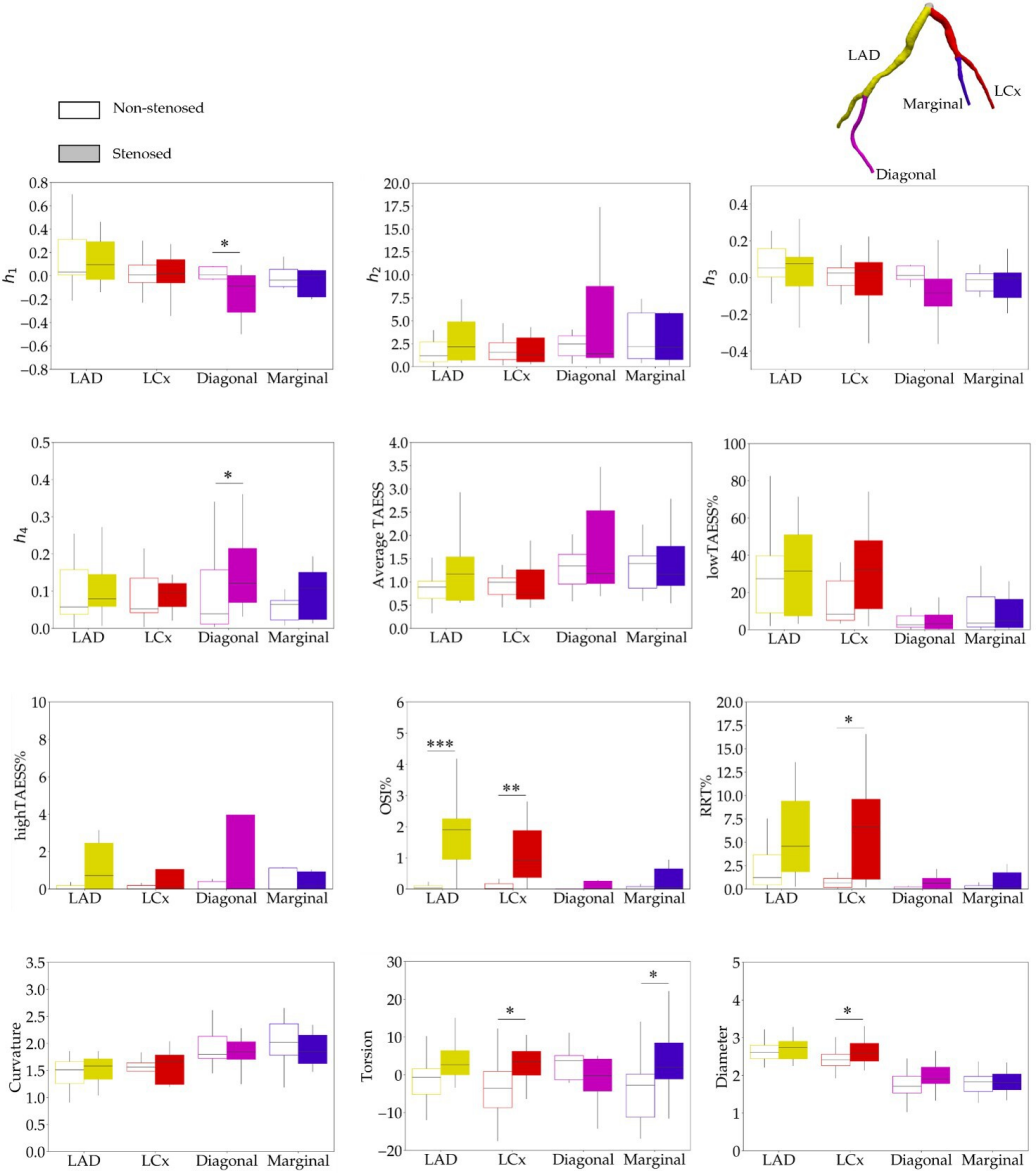
Boxplots illustrating helical flow (HF) and near-wall haemodynamic parameters in non-stenosed (unfilled) versus stenosed (filled) segments of the left anterior descending (LAD, yellow), left circumflex (LCx, red), diagonal (pink) and marginal (blue) coronary arteries. Statistical comparisons were performed using Welch’s *t*‐test for normally distributed variables and the Mann–Whitney *U*-test for non-normally distributed variables, with a significance threshold of *p* < 0.05. Statistically significant differences are denoted by asterisks (*p < 0.05, ^∗∗^*p* < 0.01, ^∗∗∗^*p* < 0.001).

The vessel-specific comparison between non-stenosed and stenosed groups revealed a significant difference in HF *h*_1_ (p=0.0483) and *h*_4_ (p=0.0168) only in the diagonal, whereas other coronary tree segments had similar HF (figure 3). No significant difference in *h*_2_ and *h*_3_ existed between all stenosis-free segments from both groups.

No significant differences were found for average TAESS, lowTAESS% or highTAESS%. OSI% was significantly higher in stenosed LAD and LCx cases, whereas RRT% was significantly higher in LCx (figure 3, OSI% LAD:p<0.0001, non-stenosed groups: 0.03 [0.01−0.11] versus stenosed group: 1.90 [0.95−2.25]); OSI% LCx: *p* = 0.0008, non-stenosed groups: 0.02 [0.00−0.17] versus stenosed group: 0.92 [0.37−1.87]; RRT% LAD: *p* = 0.0185, non-stenosed groups: 1.23 [0.44−3.66] versus stenosed group: 4.56 [1.84−9.40]; RRT% LCx:p=0.0323, non-stenosed groups: 0.65 [0.17−1.13] versus stenosed group: 6.61 [1.04−9.59]).

Considering the geometrical factors (figure 3), the diameter (p=0.0411) and torsion (p=0.0238) of the LCx artery were significantly larger in stenosed cases compared with non-stenosed cases. Additionally, the marginal torsion was also greater in stenosed cases (p=0.0425).

### 3.2. Correlations between helical flow-based, endothelial shear stress-derived and geometrical factors in stenosis-free segments

When considering the effect of HF in stenosis-free segments in stenosed and non-stenosed trees, in both non-stenosed and stenosed cases, *h*_2_ positively correlated with the average TAESS in all the segments (p<0.05), indicating that the higher absolute helicity, *h*_2_, is associated with higher absolute TAESS across arterial segments if no disease is present locally, irrespective of disease globally. *h*_1_, *h*_3_ and *h_4_* showed minor effects on haemodynamics in all arterial segments.

In trees with global stenosis, *h*_2_ significantly correlated to lowTAESS% in all segments if no disease was present locally (LAD: ρ=−0.86,p=0.0015, LCx: ρ=−0.89,p=0.0002, diagonal: ρ=−0.80,p=0.0098, marginal: ρ=−0.94,p<0.0001). *h*_2_ also correlated with highTAESS% in LAD, LCx and diagonal (LAD: ρ=0.85,p=0.0026, LCx: ρ=−0.76,p=0.0322, diagonal: ρ=−0.84,p=0.0021).

However, in trees without any stenosis, *h*_2_ positively correlated to highTAWSS% in LAD (ρ=0.72,p=0.0212). No other correlations were found between *h*_2_ and haemodynamics in any segments, indicating a minor effect of *h*_2_ on the lumen area exposed to adverse haemodynamics in non-stenosed cases.

When considering the effect of geometrical factors in stenosis-free segments from stenosed and non-stenosed cases, only the diameter of diagonal arteries negatively correlated with average TAESS in non-stenosed cases (ρ=−0.85,p=0.0046) and *h*_1_ in the stenosed cases (ρ=−0.75,p=0.0361). No other significant correlations were found.

### 3.3. Correlations between helical flow-based, endothelial shear stress-derived and geometrical factors in stenosed segments

Within the stenosed arteries, *h*_2_ positively correlated with highTAESS% (ρ=0.87,p=0.0004), average TAESS (ρ=0.96,p<0.0001) and negatively correlated with lowTAESS% (ρ=−0.89,p=0.0001). In pre-stenosis segments, only *h*_2_ positively correlated with average TAESS (ρ=0.84,p=0.0444), while no significant correlations were observed between other HF-based descriptors and ESS-derived factors.

**Figure 4.**
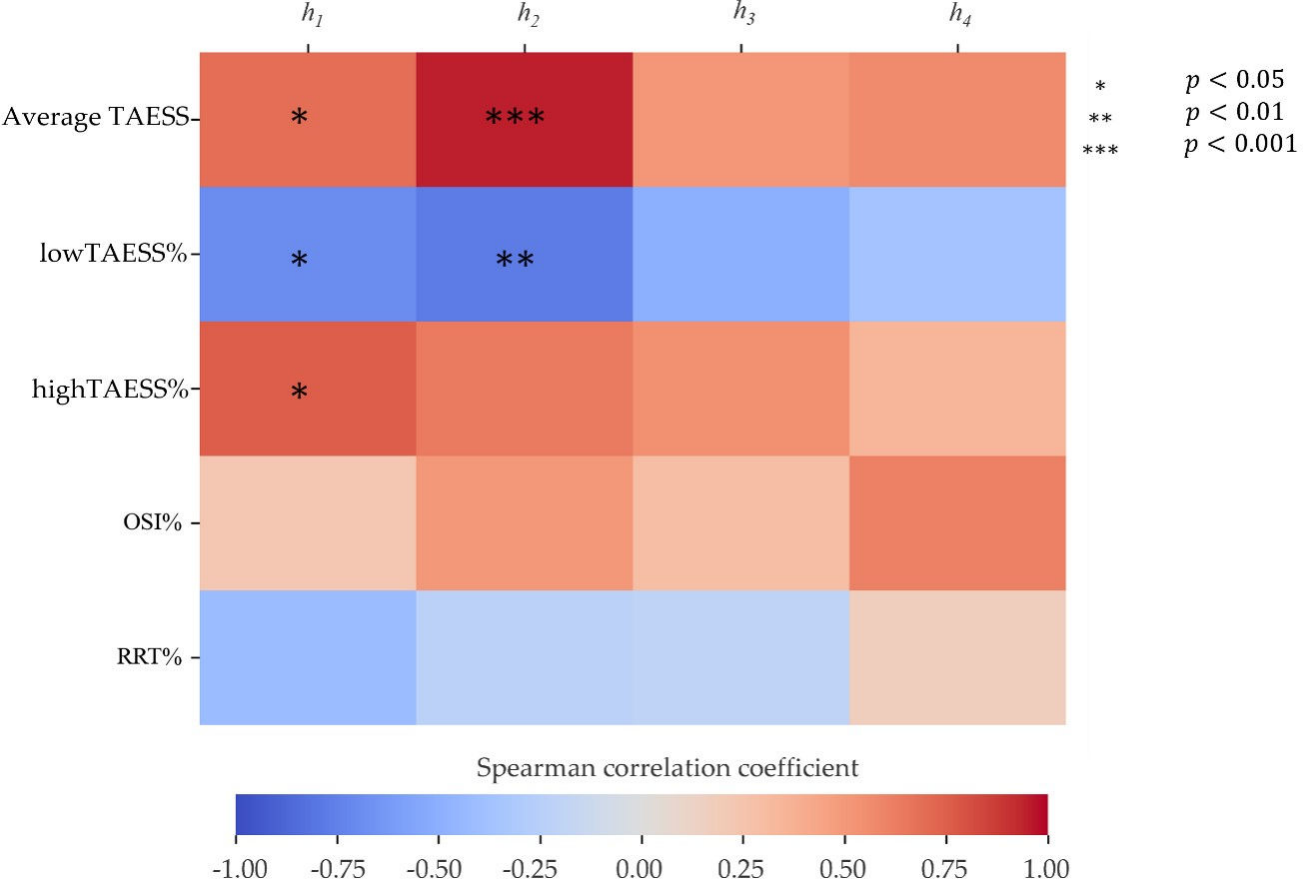
Heatmap showing the Spearman correlation coefficients between haemodynamics and HF-based descriptors in post-stenosis segments. The horizontal colour bar represents the Spearman correlation coefficient scale. Statistically significant correlations are indicated by asterisks. *h*_1_ quantifies the helicity intensity considering the rotational directions of the counter-rotating HF, and *h*_2_ measures the absolute helicity. Only helicity intensity (*h*_1_ and *h*_2_) showed significant correlations with haemodynamic descriptors. The signed (*h*_3_) and unsigned (*h*_4_) helical rotation balance did not show significant correlations with ESS-derived factors.

**Figure 5.**
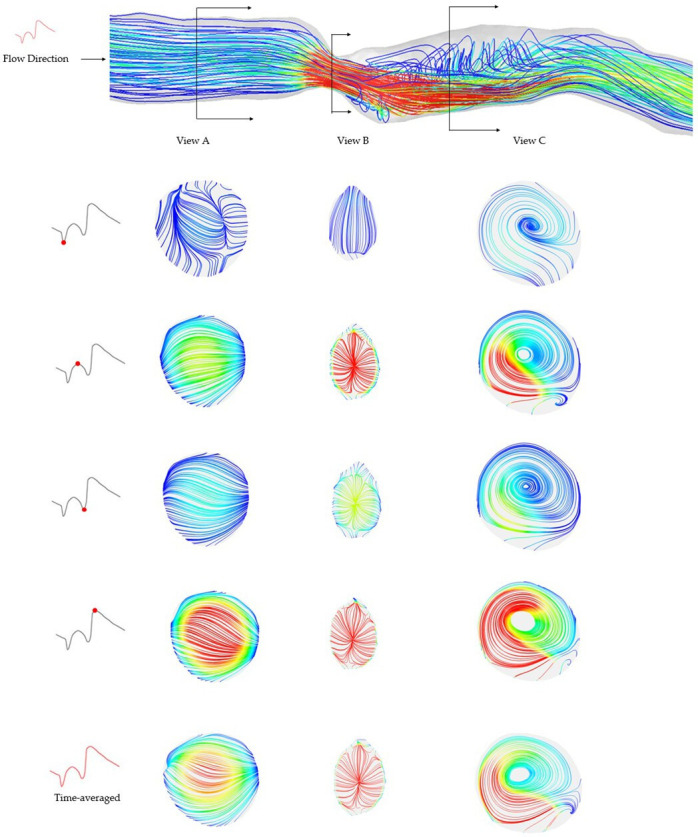
Vortex structures at different sites relative to the stenosis during a cardiac cycle. Throughout one cardiac cycle, no vortices were observed proximal to (A) or within (B) the stenosis. Vortices were present distal to the stenosis (C) due to blood flow recirculation.

The presence of stenosis significantly altered the local flow conditions (figure 2), thereby HF showed significant correlations with both low and high TAWSS. Figure 4 illustrates the Spearman correlation coefficients ρ between HF-based and ESS-derived factors in post-stenosis segments. Within and after the stenoses, jet flow induced high HF and high TAESS (figure 2, right), resulting in strong correlations between *h*_2_ and highTAESS% at the stenosis (ρ=0.86,p=0.0016), and between *h*_1_ and highTAESS% after the stenosis (ρ=0.75,p=0.0106). Due to the imbalance of the HF after the stenosis (figure 2, right), the signed *h*_1_ can better quantify the HF intensity, which explains that there is no significant correlation between *h*_2_ and highTAWSS%. Blood flow recirculation appeared immediately after the stenosis (figure 2, right), leading to vortex structures in low-velocity zones (figure 2, view C) and resulting in low TAESS distribution in the corresponding regions. Both high-signed (*h*_1_) and unsigned (*h*_2_) helicity intensity showed negative correlations with lowTAWSS%, indicating that high-intensity HF mitigated the flow disturbance after stenosis. Although imbalanced HF was observed in the post-stenosed segment regions (figure 2, right), *h*_3_ and *h*_4_, quantifying the HF imbalance, did not show significant correlations (figure 4).

## 4. Discussion

In this work, we investigated the influence of HF on haemodynamics and considered the coronary geometrical effect in 39 patient-specific artery trees using the open-source ASOCA dataset (20 non-stenosed versus 19 stenosed left coronary arteries) [31,32]. Consistent with previous findings [18,19], HF with high absolute intensity (*h_2_*) correlated with an increase in TAESS in both non-stenosed and stenosed arteries, thereby reducing the arterial areas exposed to adversely low TAESS. However, we demonstrated for the first time that this also increases arterial areas exposed to adversely high TAESS in stenosed arteries.

Previous studies have reported that stenosis increases local HF intensity [11] and TAESS [29,30]. However, the plaque can further progress or even rupture around the stenosis, making the high-intensity HF’s effect unclear. Our results indicated that high-speed and high-intensity HF in stenosed regions negatively affects the vessel wall, leading to excessively high TAESS. This has been linked to exacerbated plaque vulnerability, leading to destabilization and rupture [21,22], thereby increasing the risk of severe cardiac events [24]. At the same time, high-intensity HF can still mitigate exposure to low TAESS, which is caused by flow disturbances, vortices and low-velocity zones due to recirculation. Consequently, high-intensity HF in stenosed segments demonstrates a double-edged nature. While it reduces luminal areas exposed to adversely low TAESS, it also increases adversely high TAESS in stenosed regions. Therefore, it can be argued that for already diseased cases, more emphasis should be placed on the adverse impact of localized high-intensity HF on existing plaque.

The positive correlation between HF intensity and high TAESS in stenosis-free segments in stenosed groups can partially explain the previous findings that SCAD [15] and non-obstructive myocardial ischaemia [16,17] observed in curved arterial segments, where high HF intensity was observed [10–12]. Both SCAD [15] and non-obstructive myocardial ischaemia [24,25] have been linked to adversely high TAESS. In fact, SCAD was co-localized with higher TAESS (90th percentile) in extremely curved segments, and vessels with healed SCAD showed lower TAESS peak values [15], which indicates the effect of adversely high TAESS on SCAD development. Besides, compared with low TAESS, which is associated with decreased lumen area (constrictive remodelling), adversely high TAESS is related to expansive remodelling with high plaque vulnerability [20,22]. Although we did not examine the HF in coronary arteries with SCAD or non-obstructive myocardial ischaemia, the association found between high HF intensity and adversely high TAESS can help to explain the apparent contradiction between the reported protective effects of high HF intensity [19,26] and the clinical outcomes observed in severely curved arteries [14–17]. This relationship also provides new insight into disease development in severely curved segments by linking geometry-induced high HF intensity with the risk of excessively high TAESS, warranting future studies.

Interestingly, no correlations between HF intensity and high TAESS were found in the non-stenosed group. In normal arteries, HF naturally and effectively regulates flow and maintains TAESS within a healthy physiological range. However, in arteries with complex geometries, such as severe curvature, or after the disease has onset, overly increased HF intensity can lead to adverse haemodynamics, potentially influencing disease progression. This is in line with recent findings, highlighting the different effects of haemodynamic metrics according to disease stage [9]. Therefore, it is crucial to focus on the relationship between HF and adversely high TAESS in vessels with existing disease or complex geometries to better understand the role of HF in the development of atherosclerotic coronary artery disease.

This study has some limitations. Although 39 coronary artery trees were considered in this work, which included more patient-specific geometries than previous studies (*n* = 3), the distributions of measured geometrical factors showed outliers, indicating individual differences. Consequently, no statistically significant correlations were found between geometrical and HF-based factors, which was reported in previous studies [10–12]. Additionally, the use of Bonferroni–Holm correction in our statistical analysis, aimed at minimizing the type I error [47], may have contributed to this lack of correlation, as this correction was not applied in previous studies. In fact, before applying the correction, significant correlations were observed between geometrical factors and haemodynamic descriptors, suggesting a potential effect of geometrical factors. Thus, studies using larger datasets will be beneficial to HF studies, covering individual variations among the population. Therefore, future studies with larger datasets are warranted to account for individual variability and to further explore geometrical effects on HF.

Another limitation of this study is the assumption of rigid vessel walls, which neglects the effects of wall compliance and arterial motions. However, previous studies comparing CFD and fluid–structure interaction (FSI) have reported that wall compliance has a minor impact on the time-averaged haemodynamic parameters, such as TAESS [48–50]. Additionally, realistic capture of cardiac motion requires dynamic imaging modalities, such as invasive coronary angiography, which were not available in the present study. Moreover, the application of FSI could introduce further uncertainty due to challenges in accurately defining material properties [51]. Therefore, the use of CFD with rigid walls in this work remains a justified and widely adopted approach in relevant studies [2–5,18,19].

Due to the unavailability of *in vivo* flow measurements, a generic boundary set-up was adopted as in the previous literature. Scaling laws were used to estimate the inflow rate, and a flow-split strategy was applied to the outlets [35], which were empirically modified from Murray’s law with a robust correlation between flow and diameter at the inlet and the flow-split ratio between daughter branches compared with *in vivo* measurements [35]. Prior work has shown that the flow-split outlet strategy yields haemodynamic results comparable to multi-scale modelling under resting conditions in coronary arteries across stenoses of varying severity [36]. In this study, we examined coronary haemodynamics under the resting condition; therefore, the use of scaling laws for inflow and the flow-split strategy at the outlets provides an effective boundary condition in the absence of *in vivo* measurements.

## 5. Conclusion

In conclusion, our study provides new insights into the relationship between HF and adversely high TAESS by analysing patient-specific non-stenosed and stenosed coronary arteries. HF with higher *h*_2_ in stenosed regions correlated with a larger distribution of adversely high TAESS, which is linked to increased plaque vulnerability and a higher risk of cardiac events. Although high-intensity HF can mitigate flow disturbance by increasing adversely low TAESS in both non-stenosed and stenosed coronaries, it can at the same time cause TAESS to exceed the healthy physiological range, especially in stenosed or complex segments (with severe curvature or similar), potentially exaggerating disease processes associated with adversely high luminal shear stress.

## Data Availability

The image data used is publicly available through the UK Data Service [32]. The statistical analysis results are available in the electronic supplementary material [52].

## Appendix

See tables 2 and 3 and figure 5.

**Table 2.** Correlations between geometrical and haemodynamic factors of each segment from healthy cases with initial or adjusted *p* < 0.05.

segment	factor 1	factor 2	coefficient	initial *p*-value	adjusted *p*-value
LAD	torsion	RRT%	−0.46	0.0487	1.0000
LCx	torsion	*h*_1_	−0.50	0.0361	1.0000
diagonal	torsion	*h*_1_	0.65	0.0114	0.5009
	torsion	*h*_3_	0.56	0.0389	1.0000
	diameter	*h*_2_	−0.62	0.0186	0.7814
	diameter	average TAESS	−0.85	0.0001	0.0046
	diameter	highTAESS%	−0.62	0.0169	0.7255
marginal	diameter	lowTAESS%	0.70	0.0039	0.1804
	diameter	average TAESS	−0.68	0.0054	0.2437
	diameter	RRT%	0.61	0.0155	0.6367
	curvature	lowTAESS%	−0.60	0.0172	0.6880

**Table 3.** Correlations between geometrical and haemodynamic factors of each diseased-free segment from diseased cases with initial or adjusted *p* < 0.05.

segments	factor 1	factor 2	coefficient	initial *p*-value	adjusted *p*-value
diagonal	diameter	*h*_1_	−0.75	0.0008	0.0361
	diameter	*h*_3_	−0.55	0.0273	1.0000
	curvature	*h*_1_	0.57	0.0218	0.9162
	curvature	*h*_3_	0.57	0.0218	0.9162
marginal	diameter	RRT%	0.6203151	0.0179	0.7716
